# Dual-career student athletes in Spanish universities: characteristics and interests

**DOI:** 10.3389/fspor.2024.1507859

**Published:** 2025-01-06

**Authors:** Carlos Hernando Domingo, Marta Renau-Michavila, María Pilar Marín Gil

**Affiliations:** ^1^ Sports Service, Department of Education and Specific Didactics, Universitat Jaume I, Castellón, Spain; ^2^ Sports Service, Department of Translation and Communication, Universitat Jaume I, Castellón, Spain; ^3^ Sports Service, Universitat Jaume I, Castellón, Spain

**Keywords:** dual career, elite athletes, higher education, benefits, Spanish universities

## Abstract

The path that student-athletes follow to pursue their dual careers is both complex and demanding. However, an increasing number of elite athletes enroll in higher education today. To explore the current situation of elite university athletes in Spain, a study was conducted with the main objective of identifying their characteristics, concerns, interests, and challenges when combining their sporting and academic careers. To this end, a survey consisting of 27 items was distributed among a group of Spanish universities belonging to the Spanish University Sports Committee. A total of 563 responses were collected, of which 411 were ultimately processed from 27 different Spanish universities, providing us with insight into pursuing a dual career in Spanish universities. Descriptive data were gathered on various aspects such as age, field of study, hours dedicated to sports and studies, sports level, most relevant sports disciplines, interest in transnational mobility, and the perception and use of the benefits student-athletes receive when pursuing a dual career. Ultimately, this work aims to assist higher education institutions in developing tools that facilitate the program design for elite athletes.

## 1 Introduction

The dual career of elite athletes has been studied for decades. The balance between training, peak athletic performance, and the professional prospects at the end of the athletic career have been topics of great interest (1–4).

All this interest revolves around the emergence of the term “dual career” (DC). This term was coined with the publication of the European Commission's White Paper on Sport in 2007 (5–7) and has become a key element in promoting actions aimed at improving the well-being of this group (6, 8). A balance is sought in integrating a sporting career with education or work during the years of maximum sporting activity (9–11). In 2012, the European Commission itself published a guide to promoting dual careers, providing a framework for actions to strengthen them (12).

Based on the definitions of DC athletes, which consider both the work and education framework together with the sport framework (13–16), it is necessary to specify that this work is circumscribed within the framework of elite university student athletes who are studying at the highest level of their sporting careers.

This stage is a very complex period and has a significant impact on this group, which maintains a dual profile of student and athlete. This fact has been demonstrated in various publications, which show the difficulty of combining higher level academic training with a sports career (1, 13, 17–19). It has been identified as a period of high stress that directly affects the development of the dual career and the psychological well-being of the person (13, 20, 21). However, there are studies that demonstrate a correlation between motivation (22) and identity (23) and the level of competition and age of the student athletes in question. These studies indicate that the values in question are higher in those student athletes who have a higher level of sport and are younger.

One aspect that has been highlighted, and that needs to be considered, is the concern about career opportunities: how to facilitate the transition to the labor market when they finish their peak performance phase and retire from their sports career. In this regard, it is necessary to account for the duration of the sporting career and its stages, as well as the fact that a higher level of academic training contributes to a better transition (2, 24–27). Thus, programs that facilitate the combination of higher education with a balanced athletic career ensure a smoother transition to the labor market at the end of the athletic career (28–30).

This reality has been reflected at the European level in different intervention models: one where the government acts as regulator or facilitator; another carried out by national sports federations; and a third with no specific structure (1, 15, 31, 32).

Spain falls into the first category, in which the government acts both as regulator and facilitator. National laws affecting elite, high-performance, and high-competition athletes (33–37) exist, along with regional laws that guarantee aid to this group of student athletes.

In addition to these regulations, the autonomy of universities allows for the expansion of support programs for this group.

In this context, the Spanish government, through the Consejo Superior de Deportes, manages the Programa de Atención al Deportista de Alto Nivel (PROAD) (https://n9.cl/1fw1p), which aims to promote a policy of support and comprehensive care for all athletes. According to data provided by PROAD (38), 53 universities claim to offer some kind of support to elite athletes. In addition to government regulations, universities are taking steps to achieve a balance between academic training and athletic careers. Ten years ago, López de Subijana (39) analyzed the participation of universities in processes to promote this balance between studies and sports careers from the point of view of what students receive from the university. It is necessary to understand their perceptions and interests, and also to determine if students utilize what the university offers or if they require other types of help.

Thus, the main aim of this paper is to identify the current situation of elite university athletes in Spain, their characteristics, concerns, interests, and challenges in combining their sporting and academic careers.

## 2 Materials and methods

This study is based on a European study on DC student athletes (40), conducted by the Student Athletes Erasmus+ Mobility in Europe (SAMEurope) consortium and funded by the European Commission (Erasmus+ project 101050378).

### 2.1 Subjects

The survey, which was conducted from March 1 to May 12, 2023, involved 46 Spanish universities that are members of the Spanish Committee of University Sport and that had participated in a previous study on dual careers in Spain. Of the 46 universities invited to participate in the study, DC student athletes from 27 universities responded to the survey.

### 2.2 Procedures

The Student Athletes Erasmus+ Mobility in Europe (SAMEurope) project on DC student athletes identified and validated 31 benefits offered by the consortium universities to DC students. The project consortium then surveyed DC experts and DC students at the consortium universities. The survey process is described in Hernando et al. (40).

To collect information on elite university student athletes in Spain, the survey developed by SAMEurope was adapted to the Spanish context, using the Qualtrics® program. This research was approved by the Ethics Committee of Universitat Jaume I of Castellón (approval number CEISH/27/2022 of 21-12-2022 obtained by written document). It complies with the criteria laid down in the Declaration of Helsinki.

All participants were university students aged 18 years or older. No minors were involved in the study. In the first question of the survey, participants received full information about the project and the safety conditions were explained. Participants who were willing to take part in the survey gave a written consent to participate in the first question and could then complete the survey. If participants refused to participate, they could not answer any further questions in the survey.

The survey can be found in Supplementary Material 1.

**Figure 1:**
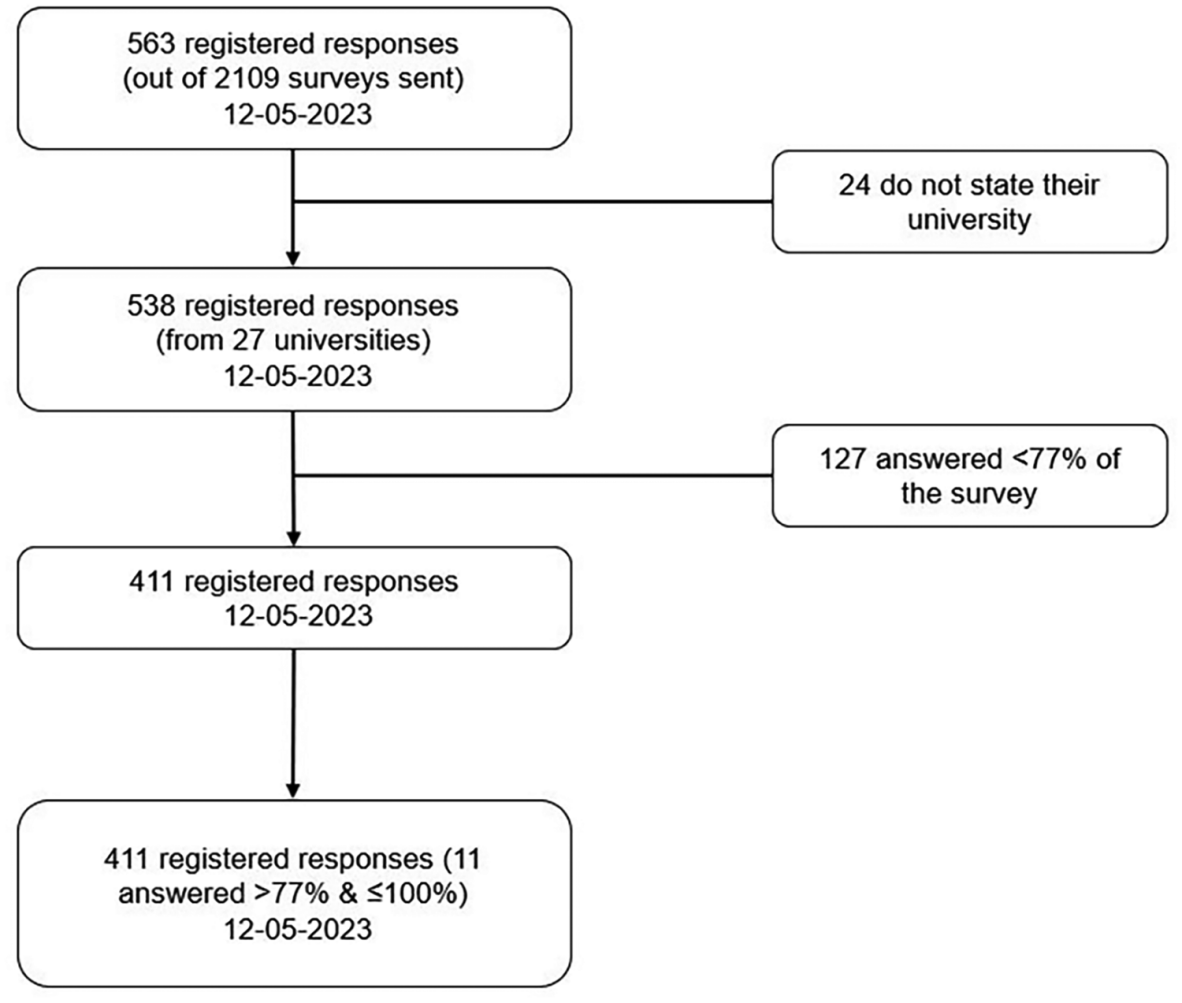
Response flow.

The surveys were sent to the DC students in the following order. First, the link to the Qualtrics® survey was sent to the people responsible for dual careers in the 46 Spanish universities (out of the 78 universities that are members of the Spanish Committee of University Sport) that had participated in a previous study on the DC situation in Spanish universities. Second, each university sent the link to its DC student athletes to complete the survey. The survey was available from March 1 to May 12, 2023. A total of 563 responses were collected, of which 411 responses from 27 Spanish universities were processed, as shown in Figure 1.

The survey included seven sections:

1. Consent (question 1).
2. Personal information: year of birth and gender (questions 2–3).
3. Academic data: country where you are studying, name of the university where you are studying, level of study, field of knowledge, name of the degree you are studying, number of credits enrolled in this academic year, and hours per week devoted to university studies (questions 4–10).
4. Sports data: type of sport you participate in, sport modality, level of sport, highest level of championship in which you participated, hours per week spent on sports, and participation in university competitions (questions 11–16).
5. Dual career: criteria for entering the university, criteria for choosing the university, problems in combining elite sport with studies, evaluation of the importance of the benefits offered by the university for combining university studies and elite sport, use of the benefits offered by being a DC student, list of the benefits used and support persons in the dual career (questions 17–24).
6. International mobility: interest in doing an Erasmus+ stay and criteria for choosing a host university (questions 25–26).
7. Recognition of the dual career (question 27).

In the dual career section, among other questions, student athletes were asked to rate the 31 benefits listed in the SAMEurope project and collected by Hernando et al. (40). Question Q21 asked DC student athletes to rate the importance of each of the 31 benefits of combining university studies and elite sport from 0 to 5 (0 = not at all important, 5 = very important). In question Q23, they were asked to indicate which benefits they had taken advantage of as DC students.

With the data obtained from these two questions, the value of the benefit and the number of times each benefit was used, a calculation formula was created to combine the two results and assess the relevance of each benefit. Specifically, the level of importance of a benefit (LI) was multiplied by the percentage of use of this benefit [the number of uses of the benefit (NU) divided by the total number of responses received (TAS), in this case 411], and the result was multiplied by 10 to obtain a number that was not too small [LI × (NU/TAS) × 10]. This calculation makes it possible to rank the benefits by combining the importance for the students and the number of uses of the benefit in relation to the total number of participating students. The ranked benefits were then divided into quartiles to identify those benefits that were in the first quartile, and those that were in the second, third, and fourth quartiles.

Then, to determine the relationship between the importance that the students attached to each benefit and the number of times it was used, a Pearson correlation was performed between the two variables, level of importance and number of times each benefit was used.

### 2.3 Data analysis

A descriptive study on the situation of dual careers among Spanish university students was carried out. Based on the work carried out by López de Subijana et al. in 2014 and 2015 in Spanish universities (13, 39), a sample calculation was performed to obtain a confidence level greater than 95% with a margin of error of ±5%. Statistical analysis was performed using SPSS v27 software, and results were considered statistically significant if the *p*-value was less than 0.05. The Kolmogorov-Smirnov test was used to test the normality of the data. Data were presented as mean and standard deviation for continuous variables. For frequency or proportion variables, sample size values were used. The distribution of benefits was divided into quartiles using Tukey's hinge model. When the data did not meet normality criteria, a nonparametric statistic was used. The Kruskal-Wallis test was used with a Mann Whitney *post hoc* test for pairwise comparisons. The Pearson correlation was used to analyze the correlation between the evaluation of the benefits and their use by the students. Cohen's criterion was used as the framework in which the degree of correlation was determined, with a weak correlation when the value is between 0.1 and 0.29, a moderate correlation when the value is between 0.30 and 0.50, and a strong correlation when the value is higher than 0.50 (39).

## 3 Results

The survey was distributed to 46 Spanish universities, which in turn sent the link to the survey to 2,109 students. A total of 411 students participated, which corresponds to a participation rate of 19.49%. The results have a confidence level of more than 95% and a margin of error of ±5%.

Table 1 shows the names of the participating universities and the number of links to the survey that each university emailed to its DC students, sorted by university (universities marked with an asterisk did not provide information on the number of DC students to whom they sent the survey link), as well as the number of accepted responses and the percentage of participation they represent.

**Table 1:** Number of surveys sent and accepted and% they represent.

	Sent	Accepted	%
Universidad Carlos III de Madrid	86	25	29,07%
Universidad Católica San Antonio de Murcia	218	41	18,81%
Universidad Católica San Vicente Mártir de Valencia	14	2	14,29%
Universidad de Alcalá	43	11	25,58%
Universidad de Alicante	131	13	9,92%
Universidad de Almería	27	6	22,22%
Universidad de Barcelona	147	42	28,57%
Universidad de Burgos	19	7	36,84%
Universidad de Deusto	5	1	20,00%
Universidad de Girona	74	9	12,16%
Universidad de Huelva	7	2	28,57%
Universidad de las Islas Baleares	53	3	5,66%
Universidad de Murcia	76	12	15,79%
Universidad de Navarra	*	2	*
Universidad de Valencia	322	20	6,21%
Universidad de Valladolid	264	33	12,5%
Universidad de Vigo	71	24	33,80%
Universidad Miguel Hernández	26	5	19,23%
Universidad Nacional de Educación a Distancia (UNED)	*	1	*
Universidad Pablo de Olavide	82	8	9,76%
Universidad Pública de Navarra	74	22	29,73%
Universidad San Pablo CEU	40	7	17,50%
Universitat Autònoma de Barcelona	*	1	*
Universitat Internacional de Catalunya (UIC Barcelona)	38	10	26,32%
Universitat Jaume I de Castelló	95	50	52,63%
Universitat Politècnica de València	120	37	30,83%
Universitat Pompeu Fabra	77	17	20,08%
Total	2,109	411	19,49%

* Data not provided.

A total of 411 DC students participated in the study, of whom 207 were female, 202 were male, and 2 identified as non-binary. The majority of these students, 388, were pursuing graduate studies, 17 were enrolled in master's programs, and 6 were engaged in doctoral studies.

The students who responded to the survey had a mean age of 21.53 ± 3.14 years, ranging from 18 to 41 years, with the vast majority (79.08%) between the ages of 19 and 22.

As stated above, 94% of the students are enrolled in undergraduate programs, 4% are enrolled in master's programs, and 2% of the DC students who responded to the survey are enrolled in doctoral programs.

The field of knowledge proposed by UNESCO was established using a two-digit system to identify the field in which all dual career students were studying (41). It is evident that 25% of the dual career (DC) students are pursuing studies in the field of medical sciences, followed by 16% in technological sciences, 14% in economic sciences, and 12% in life sciences. The total number of fields of study pursued by these student athletes can be found in Supplementary Material 2.

Table 2 shows the results obtained from the sports profile of the DC students who responded to the survey, including credits enrolled, hours spent studying and practicing sport on a weekly basis, as well as participation in National University Championships.

**Table 2:** Sport profile.

		Type of sport		Sport level				Highest Int. championship					
	ALL	NA	A	R	N	Int	K-W	OG	WC	EC	IUC	NT	K-W
N	411	378	33	22	243	146		5	83	31	4	23	
CE	57.10 ± 13.72	56.87 ± 13.91	59.70 ± 11.09	60.11 ± 12.78^a^	58.51 ± 13.72^b^	54.30 ± 14.77^a,b^	**0.003**	56.8 ± 14.81	53.29 ± 15.17	51.97 ± 16.05	57.00 ± 8.12	60.09 ± 12.87	0.112
H St/W	27.25 ± 11.05	27.32 ± 11.02	26.48 ± 11.66	27.79 ± 10.81^a,b^	33.27 ± 8.35^a,c^	25.45 ± 11.46^a,b,c^	**0.002**	15.80 ± 11.02^x,y^	24.60 ± 11.11^x,z^	30.29 ± 11.41^y,z^	25.75 ± 18.41	24.00 ± 10.75	**0**.**039**
H Sp/w	20.47 ± 8.84	20.67 ± 8.88	18.18 ± 8.30	13.36 ± 5.36^a,b^	19.14 ± 8.08^a,c^	23.76 ± 9.30^a,b,c^	**1.64E-10**	35.00 ± 9.45	23.28 ± 9.11	22.97 ± 9.05	25.25 ± 4.5	23.91 ± 9.89	0.143
P-NUC Yes (N)	242	223	19	18	159	65		1	28	19	4	13	
P-NUC No (N)	169	155	14	4	84	81		4	55	12	0	10	

Data are presented as mean and standard deviation in continuous variables, sample size is indicated in frequency variables. N, number; CE, credits enrolled; H St/W, hours study/week; H Sp/w, hours sport/week; P-NUC, participation in National University Championships; ALL, all students; NA, not adapted; A, adapted; R, regional; N, national; Int, international; K-W, Kruskal-Wallis test; OG, Olympic Games; WC, World Championship; EU, European Championship; IUC, International University Championship; NT, National Team. ^a,b,c,x,y,z^ indicate the values of significant differences between groups according to the Mann-Whitney test. Bold type indicates values indicating values with statistical significance in the Kruskal-Wallis test.

Ninety-two percent of the DC students participate in non-disabled sport modalities, but it is interesting to note that 8% of the DC students practice their high level sport in the adapted sport modality.

Regarding the distribution of students by sport level, the surveys show that 36% of DC student athletes are considered international level athletes, while 59% are considered national level athletes and 5% are considered regional level athletes.

Of the DC student athletes at the international level (146 in total), 5 have competed in Olympic Games and the majority (83) have competed in World Championships. The question (Q14) asked what the highest level of competition they had participated in was, suggesting that the highest level in this section implies that they have also participated in lower level competitions.

The number of credits enrolled is 57.10 ± 13.72 and they spend 27.25 ± 11.06 h per week on their studies. The number of hours spent per week on aspects related to sport (training, physiotherapy, medical.) is 20.47 ± 8.85 h per week. 59% of DC students participate in national university competitions. A detailed breakdown of the distribution of enrolled credits, study hours and sport hours by profile can be seen in Table 2.

A total of 70 distinct sports modalities were reported by all respondents to the survey. The most popular sport was track and field, with 70 out of 411 respondents indicating this as their primary sport. This was followed by basketball, handball, and swimming, with 20 out of 411 respondents indicating each of these as their primary sport. A total of nine sports (five team sports and four individual sports) account for 50.37% of the dual career student-athletes who participated in the study. The remaining 49.63% are distributed among 61 different sports. Details of the sports played by the total number of dual career students who participated in the survey can be found in Supplementary Material 3.

**Figure 2:**
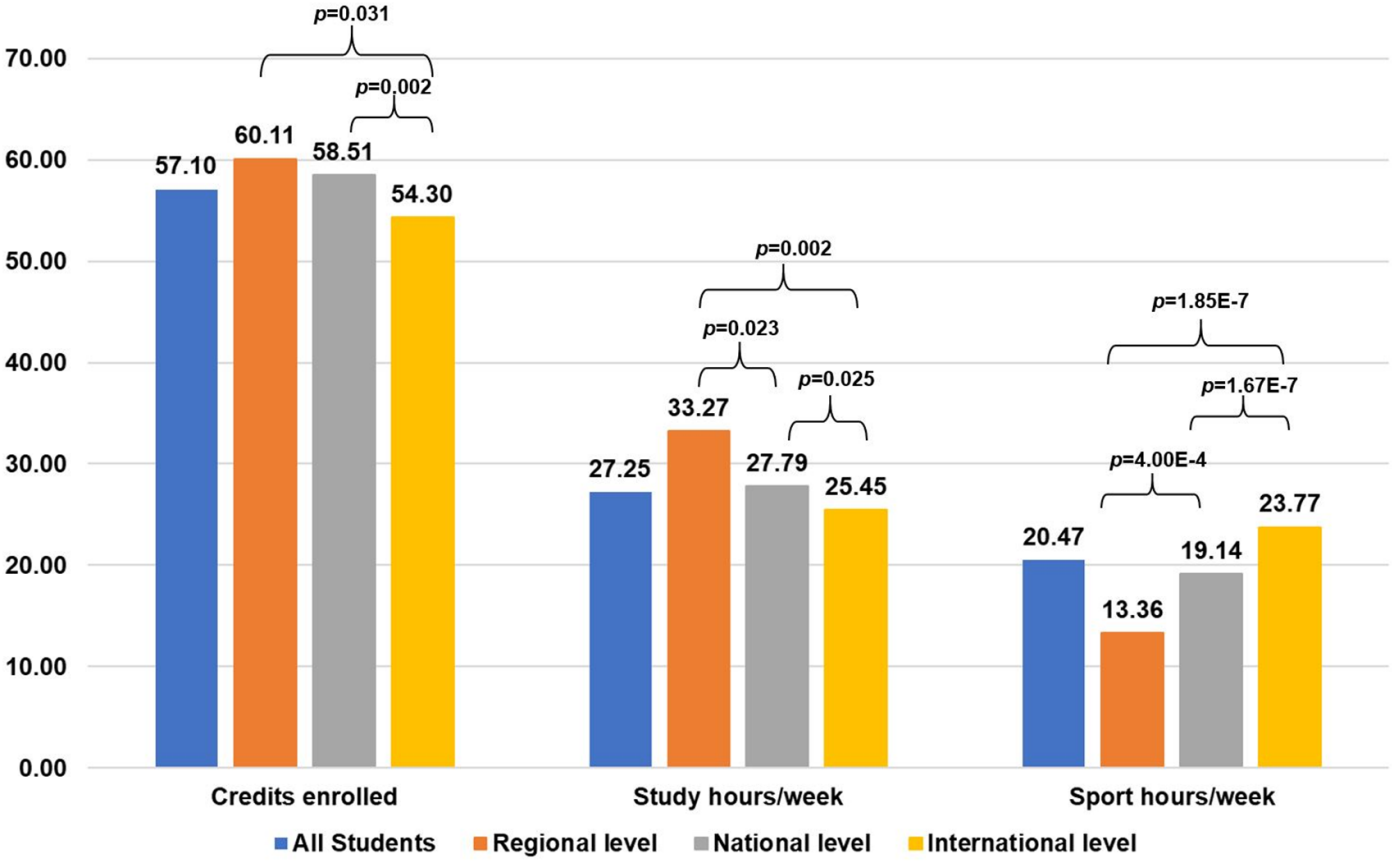
Dc students: credits enrolled and hours/week devoted to study and sport. Curly braces indicate significant paired differences between sport level groups. *p p*-value.

Figure 2 shows the results disaggregated by sport level to illustrate the differences in credits enrolled, hours of study, and hours of sport by sport level of DC student-athletes.

There are significant differences in the number of credits enrolled between the international level group and the national and regional level groups. This is replicated in hours of study and hours of sport. The regional level group also shows significant differences from the national level group in the number of hours devoted to sport and to study.

**Figure 3:**
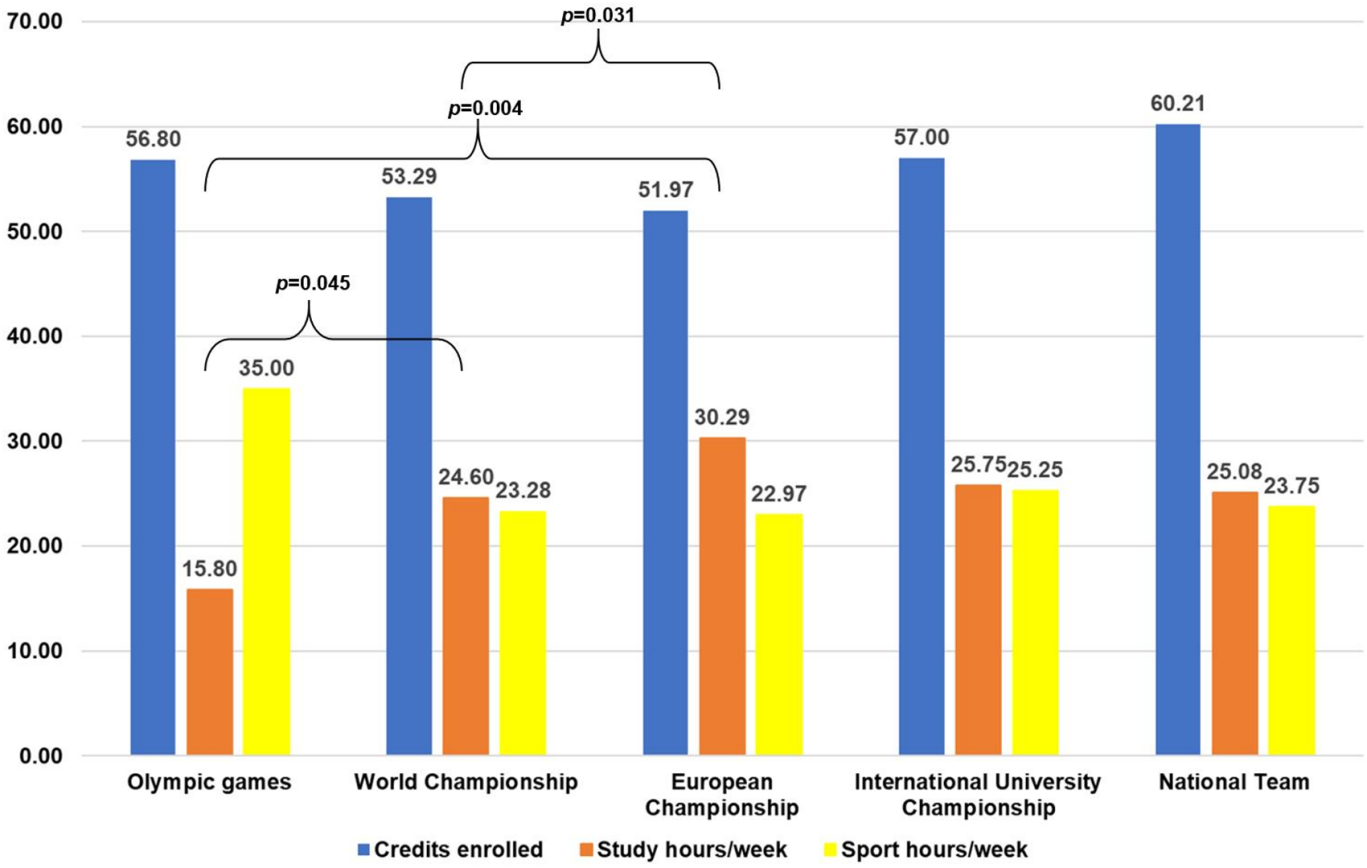
International DC students: credits enrolled and hours/week devoted to study and sport. Curly braces indicate significant paired differences between sport level groups. *p p*-value.

In Figure 3, special attention is paid to the international level DC students to examine their differences in terms of credits enrolled, hours of study, and hours devoted to sports-related aspects. When the international level group is analyzed in detail, there are no significant differences in the number of credits enrolled or hours devoted to sport among the five groups that make up this level.

However, there are significant differences in the number of hours devoted to studying, especially in the group of Olympic athletes, which is different from the group of athletes who participate in World Championships and from the group of athletes who participate in European Championships. There is also a significant difference between the athletes who participate in the World Championships and those who participate in the European Championships.

Fifty-two percent of the DC students from the 27 Spanish universities represented in the survey indicated that they entered their studies based on academic criteria, with 48% of those who entered their studies based on sports criteria regulated by national or regional regulations (Law 39/2022; RD 971/2007; D 39/2020) which reserve places for high-level, high-performance or elite athletes.

Similarly, 49% of the students stated that the choice of the university where they could pursue their higher education was based on the support that each university offered to DC students in combining their studies and their sports careers.

**Figure 4:**
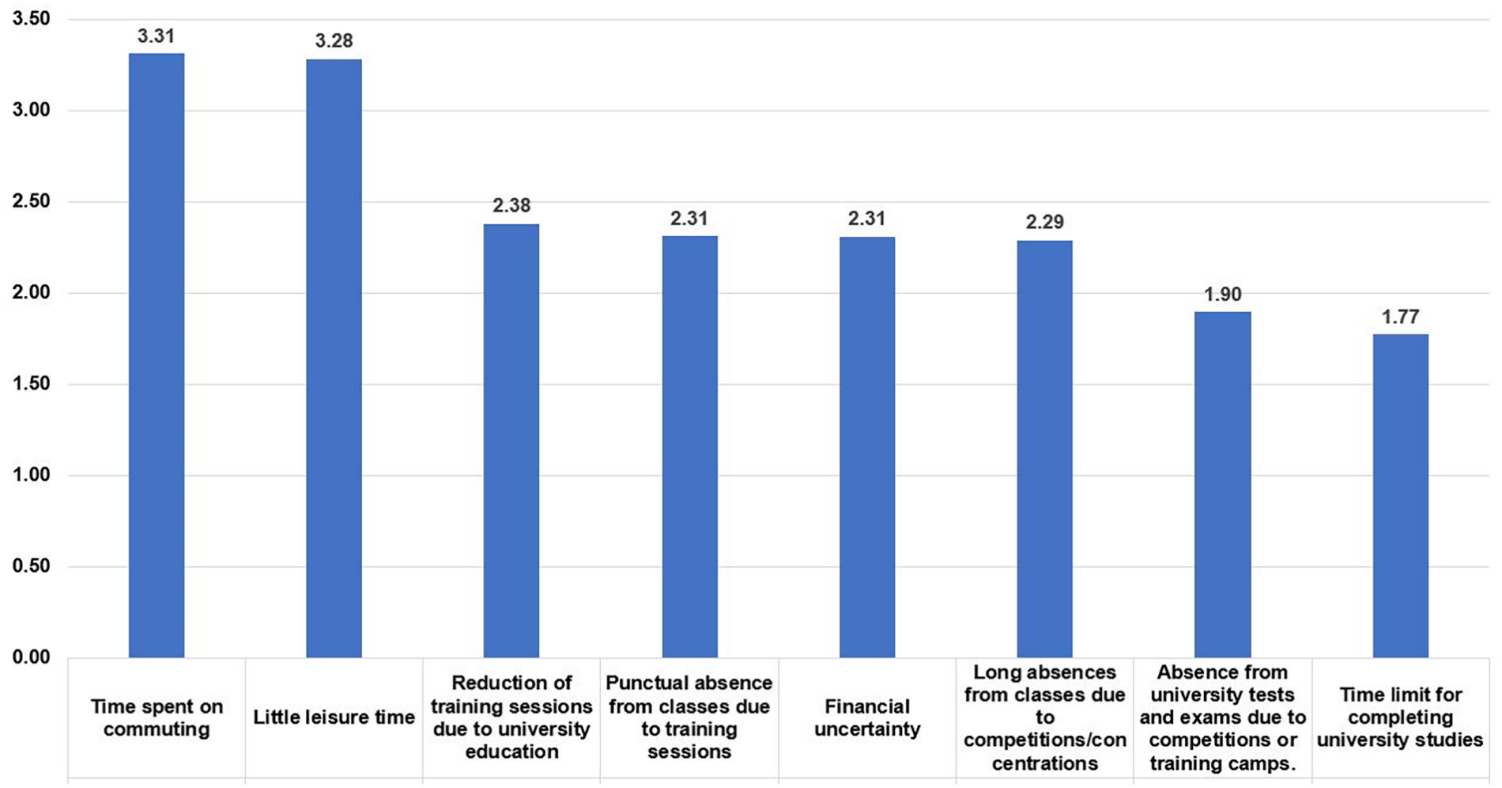
Problems in combining elite sport with studies.

Figure 4 shows the degree of importance DC students place on the problems encountered reported in combining their studies and sports careers. It can be seen that managing the time dedicated to travel and the lack of free time are the main problems for the development of their dual career.

Sixty-one percent of DC student athletes say they are aware that their university can help them balance their studies with playing a high-level sport, although 39% are not aware of this option.

DC student athletes were asked to rate a series of 31 benefits from 0 (not important) to 5 (very important).

Column LI of Table 3 shows the importance of each benefit from the students' point of view. Scholarships are the most valued benefit, followed by the change of exams and the justification of absences.

**Table 3:** Valuation of benefits by DC athletes.

	Benefits	LI	NU	LI × (NU/TAS) × 10	GO	Q
ACADEMIA	Justification for absences	4.35 ± 1.19	220	23.26	1	1
Academia	Changing exam dates	4.38 ± 1.18	149	15.87	2	1
Others	Scholarships	4.55 ± 1.04	113	12.52	3	1
Sport	Free use of sports facilities	4.15 ± 1.40	118	11.92	4	1
Academia	Choose class groups	3.74 ± 1.60	127	11.57	5	1
Academia	Academic tutoring	3.52 ± 1.56	83	7.11	6	1
Sport	Extra credit for participation in university sport events	3.90 ± 1.52	73	6.92	7	1
Sport	Private use of sports facilities	3.41 ± 1.65	49	4.07	8	1
Academia	Partial enrollment	3.14 ± 1.70	42	3.21	9	2
Health	Physiotherapy	3.93 ± 1.50	28	2.68	10	2
Academia	Adaptation of speed of study	3.65 ± 1.52	23	2.04	11	2
Sport	DC tutoring	3.16 ± 1.67	23	1.77	12	2
Sport	Reservation of places for general sports courses	3.61 ± 1.58	19	1.67	13	2
Academia	Online courses	3.22 ± 1.68	19	1.49	14	2
Academia	Career advice	3.45 ± 1.51	16	1.35	15	2
Academia	Online exams (without changing dates)	3.06 ± 1.67	18	1.34	16	2
Academia	Extension of number of exam session	3.65 ± 1.57	11	0.98	17	3
Others	Discounts in meals	3.61 ± 1.64	11	0.97	18	3
Health	Specialized PE teachers	3.53 ± 1.58	10	0.86	19	3
Others	Housing	3.25 ± 1.77	10	0.79	20	3
Health	General medical services	3.57 ± 1.61	9	0.78	21	3
Others	Extra points in Erasmus evaluation	3.70 ± 1.61	8	0.72	22	3
Health	Testing (physiology, biomechanics, performance)	3.59 ± 1.56	8	0.70	23	3
Health	Nutritionist	3.85 ± 1.46	7	0.66	24	3
Academia	Remedial courses	3.41 ± 1.55	5	0.42	25	4
Health	Mental health support	3.90 ± 1.50	4	0.38	26	4
Academia	Extension of the criteria for permanency	3.31 ± 1.65	4	0.32	27	4
Academia	Separate academic group	2.26 ± 1.67	4	0.22	28	4
Others	Adapted catering service	2.95 ± 1.74	3	0.22	29	4
Academia	Specific courses (ex. time management, sports marketing & social media)	2.52 ± 1.65	3	0.18	30	4
Academia	Free semesters	2.71 ± 1.74	2	0.13	31	4

LI, level of importance; NU, number of uses; TAS, total answers of students; GO, general order; Q, quartile. Table sorted by the GO column.

Seventy-one percent of DC students report that they have used at least one benefit. Column NU of Table 3 shows the number of times each benefit was used. The most commonly used benefit by DC student athletes is excusing absences (220 uses out of 411 respondents), followed by changing exam dates (149 out of 411) and choosing class groups (127 out of 411).

In the order of the benefits (GO column of Table 3), taking into account the two variables together —the importance of the benefit and the number of uses they have made, as indicated in the Materials and Methods section— it can be seen that the most important benefit is justification of absences, followed by change of exam dates and scholarships. The benefits are divided into four quartiles: 8 benefits in quartiles 1, 2 and 3, and 7 benefits in quartile 4.

**Figure 5:**
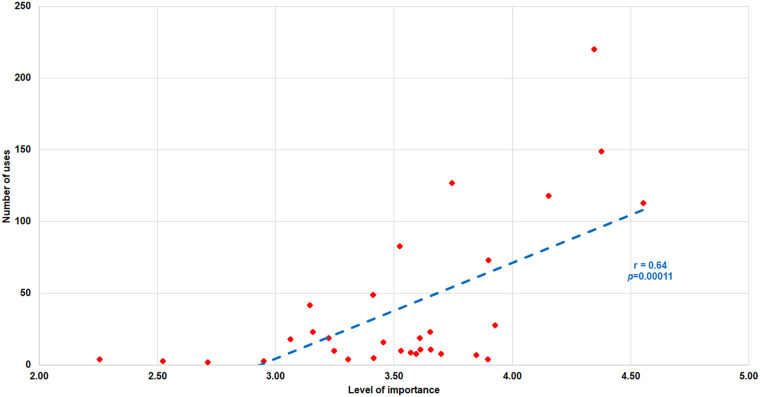
Correlation between the level of importance of the benefit and the number of times each benefit was used. *r*, Pearson correlation; *p*, *p*-value.

When analyzing the correlation between the importance of the benefit for the students and the number of uses (Figure 5), it is observed that it has a value of *r* = 0.64, a *p* = 0.00011 and a CI: 0.359–0.806. This indicates a positive, strong and significant correlation.

When students were queried about the individuals who provide them with support in pursuing their dual careers, they indicated that their classmates are the primary source of assistance, with an average rating of 3.46 on a 5-point scale. This was followed closely by other individuals, primarily family members and coaches, with an average rating of 3.37, followed by faculty or academic staff, with an average rating of 2.14, while administrative or service staff received an average rating of 2.04.

Eighty-four percent of the DC student athletes who responded to the survey said they would like to participate in an international mobility through the Erasmus+ program.

Among the criteria for choosing a destination for an Erasmus+ mobility, the first is that it offers the right conditions for developing their sports career, closely followed by the conditions for developing their university studies. Other important criteria for DC students when choosing a university abroad are the country in which the university is located and the language in which the studies are taught.

Fifty-five percent of the DC student athletes who participated in the study believe that their university does not recognize their dual career.

## 4 Discussion

The main aim of this paper is to identify the current situation of elite university athletes in Spain, their characteristics, concerns, interests, and challenges in combining their sporting and academic careers.

To this end, a number of different areas pertaining to the characteristics of DC students have been addressed. It has been determined that the average age of these students is 21.53 years old. There is an approximately equal number of men and women. The majority of them are pursuing a Bachelor's degree. There is considerable variation in the types of sports they engage in. Approximately 60% of them are concentrated in 13 sports. Similarly, the time dedicated to study and sport on a weekly basis has been identified, allowing for a comparison of the time invested in both activities with the level of sporting achievement. Furthermore, the benefits received and utilization of these have been studied in depth. In a novel addition, the mobility of these DC students has been addressed, with a high level of interest demonstrated. Throughout this section, these characteristics, perceptions and interests will be elaborated upon in depth.

In this study, 27 Spanish universities participated, with a total of 563 responses, of which only the data of 411 students were processed due to the fact that the remaining responses were incomplete (Figure 1). In the work of López de Subijana et al. (14, 39), 39 universities were analyzed to determine their support for elite athletes, with 575 of 2,378 PROAD athletes, not all of whom were university students. In 2024, according to the data provided by PROAD, 3,109 athletes have been supported by the program, of which 1,147 are pursuing higher education. Therefore, the sample analyzed in this work (411 active student athletes from 27 universities in Spain) is representative of this group in Spanish universities.

The first data point to be considered is the mean age of this group, 21.53 ± 3.14 years, which indicates that they are in a phase of mastering their sport with a high level of dedication while also pursuing higher-level studies (11, 25, 26, 42). It is also observed that the age range spans from 18 to 41 years old. There are 10 subjects, 2.43%, who are over 30 years old, which helps us understand that some athletes follow a different academic trajectory compared to other students (e.g., they start studying at the end of their sporting career, take breaks in their academic development, or extend their academic period). This phenomenon is addressed by projects focusing on the transition phase between a sporting career and integration into the labor market (29, 30, 43). The literature emphasizes that this transition is easier when higher academic education directly facilitates entry into the labor market (2, 10, 28, 44, 45). Therefore, the need to support this compatibility is a crucial action to be taken by higher education institutions.

We observed a balance between men and women, indicating a normal distribution within the group of DC students, with two subjects identifying as non-binary —a relatively new consideration in the sports world that is starting to gain recognition.

The average number of enrolled credits is 57.10 ± 13.72, nearly a full academic year, which is 60 credits, with the majority of undergraduate students (94.44%). Doctoral students represent 1.5% and Master's students 4.14%. These values indicate that some elite athletes continue their studies beyond the Bachelor's degree, which aligns with the findings of Condello et al. (17), who analyzed the increasing number of elite athletes pursuing higher academic education. This trend is recognized by the Spanish government and is reflected in Law 39/2022, of December 30, on Sport, in its Article 24.2.a, which introduces a new rule extending access to higher-level studies for elite athletes, previously limited to degrees, to Master's or postgraduate studies. However, it should be noted that the criteria for access to higher-level studies are well defined in public universities, which adhere to state and regional regulations. In contrast, private universities may have different criteria adapted to their own regulations.

Thus, Law 39/2022 of December 30 on Sports confirms the model in which the Government acts as a regulator and facilitator to support the compatibility of studies and sports careers for student athletes pursuing a dual career (15, 31, 39).

The DC students who participated in the survey, as shown above, mainly study degrees in medical sciences (24.82%), followed by technological sciences (8.76 points) and economics (over 10 points). This data contrasts with the work of Condello et al. (17), who analyzed 476 university students participating in competitions of the International University Sports Federation (FISU) and the European University Sport Association (EUSA), where the most common fields of studies were Sports Sciences, followed by Business and Administration. This work employs the UNESCO coding system, which differs from that used by Condello et al. (17). This may be a contributing factor to the discrepancies observed in the fields of knowledge pursued by DC students. For example, the field of Sports Science is not represented in the employed coding, which suggests that these studies may be subsumed within the broader domain of Medical Sciences. In Spain, in the field of Medical Sciences offers a range of degree programs that are highly sought after by DC students, including Medicine, Physiotherapy and Nursing. Furthermore, there are degrees in Sport Sciences that are offered with a health profile, which is analogous to that of Medical Sciences, and others with a Social Sciences profile, which is more closely aligned with the Life Sciences in the UNESCO codes. Moreover, the admission requirements for these programs in Spain are highly competitive, reflecting the high level of demand (46). Consequently, this group may utilize the access route stipulated in Article 9.1 of Royal Decree 971/2007, of July 13, on high-level and high-performance athletes to gain admission to these programs, given that their grades may not meet the requisite standards for direct entry. These findings appear to align with our results, as 48% of DC students who participated in the study indicated that they gained admission to university studies through the access route regulated by Article 9.1 of Royal Decree 971/2007. In addition, the total number of students pursuing studies in Medical Sciences is 25%, while the figure for those studying Life Sciences is 12%, resulting in a total of 37%.

Regarding the type of sport practiced, it is encouraging to note that 8.03% of DC students participate in adapted sports. This figure contrasts with the 1.67% of students with functional diversity who are undergraduates in Spanish public universities, according to data presented by Hernández et al. (46). This group is typically not addressed in great detail within the context of dual career in universities. By making this group visible, we can enhance their social recognition and, consequently, the likelihood of attracting new student athletes to these programs. While the percentages are low, actions that make them visible can reinforce their promotion and full inclusion and normalization within the scope of the dual career in university.

One of the important elements to point out is the complexity of reaching a consensus on the definition of an “elite athlete” in the international framework (47). Authors such as Swann et al. (48) or McKay et al. (49) have conducted studies to define more objectively the level or grade of an athlete. In Spain, the term “elite athlete” is defined in Royal Decree 971/2007, of July 13, on high-level and high-performance athletes and the criteria for being an elite athlete are well established. However, there are other athletes included in dual-career programs in universities, as Spanish universities, due to their autonomy, can go beyond the definition established by the Spanish law. In this case, 5.35% of the students are in dual-career programs at the regional level, 59.12% are at the national level and 35.52% are at the international level. The works of Condello et al. (17), with 426 athletes from 5 continents, and Capranica et al. (30), with 77 athletes from 5 countries, focus on athletes at the international level. In our case, the sample is extended to all students pursuing a dual career in Spanish universities.

For DC students at the international level, the highest percentage is for participation in world championships, with a significant number of athletes who have participated in the Olympic Games and who responded to the survey (Table 2).

Figures 2, 3 show the level of dedication to studies and sports. It is clear that the number of hours students devote to studying and sports depends on their level of sports. Thus, the higher the level of sports, the greater the number of hours devoted to sports and, in turn, the fewer the hours devoted to studies (Figure 2).

This information is relevant for understanding the distribution of time devoted to study and sport in this group of dual-career students, and it shows the differences in commitment according to their level of sport. Gjaka et al. (50) make a comparison between sport and university hours, differentiating between individual and team sports, and they show that students who participate in team sports devote a greater number of hours to university. A new line of research would be to analyze the time devoted to study and sport by sport level and to add the variable introduced by Gjaka et al. in 2024.

The findings indicate that as the level of sport participation increases, so does the amount of time spent on sport. International DC students were observed to engage in sport for 10 and 6 additional hours per week compared to regional and national students, respectively. Conversely, international DC students dedicate between two and eight fewer hours per week to academic studies than their regional and national counterparts. These findings align with those of Lupo et al. (23), indicating that the higher the level of sport, the greater its impact on the time allocated to studies. Consequently, these students require enhanced support and benefits from higher education institutions to effectively balance their academic and sporting commitments.

If we focus on the international level (Figure 3), the hours devoted to studies are quite balanced, except at the Olympic Games level, where there is a difference between the time devoted to sports and to studies, with more than twice the weekly time devoted to sports compared to studies. However, there are significant differences in the number of hours spent studying. The group of Olympic athletes devotes significantly less time to studying than athletes participating in the World Championships and those participating in the European Championships. This significant difference also occurs between students participating in the World Championships and those participating in the European Championships (Figure 3). It is important to note that the majority of Olympic athletes view athletics as a profession, which significantly influences their lifestyle. For this group, dedication to sport is akin to a full-time job (35 h per week), which poses a challenge in allocating time for academic pursuits. However, it is noteworthy that despite this lack of dedication to study (15 h per week), they are enrolled in 56.80 credits, which is equivalent to a full academic year. These results show how dedication to sport influences the possibility of devoting hours to study, so that the higher the sporting level of the student, the more they need measures to balance their academic and sporting life.

The total hours of study and sport are 46.63 h/week at the regional level, 46.93 h/week at the national level, and 49.22 h/week at the international level. If we add 56 h/week of sleep at each level, according to the sleep recommendations for adults and athletes (51–53), we have approximately 9–9.45 h/day left for their “free” time. This is reflected in Figure 5, where the DC students indicate that the issues most affecting DC athletes are clearly the time spent on transfers and their limited free time. Therefore, as in previous studies (17, 39, 40, 50, 54, 55), it is evident that time management is one of the most significant challenges for DC athletes. However, other issues also appear, such as missed training, missed classes, or financial problems. To address some of these issues, several authors (12, 29, 39, 56) advocate for distance learning as a solution. We believe that this is an interesting proposal, but it must be adapted to the profile of the university. For example, face-to-face universities, although they can use distance learning as a complementary tool, have limitations in implementing fully online studies. In contrast, universities that are already virtual or non-face-to-face by design have a model defined for offering online courses.

A total of 70 different sports modalities were detected in the set of responses, which aligns with the work of Condello et al. (17), where track and field is the most practiced sport, followed by sports such as swimming, basketball, and football. Although handball is a relevant sport in Spain, in the work of Condello et al. (17) there was no handball competition, so it is not included among the most relevant sports. Instead, baseball appears, which is not a sport with a high number of participants in Spain.

This paper examines 31 benefits offered by universities to help students manage their dual careers. The benefits analyzed are based on the work of Hernando et al. (40), which describes 31 benefits offered to varying extents in European universities. The analysis considers the level of importance assigned by this group to each benefit and whether they have used it to combine their sporting career with their academic studies. It is important to note that the benefits analyzed are those that facilitate the continuation of an academic career alongside a sporting career, i.e., they pertain to active students pursuing a university degree. Thus, benefits such as the allocation of 3% of university access places are not considered in this work, as this benefit aids access to studies, not their continuation. This distinction is important to emphasize in the context of the university because the support for DC student athletes begins after their enrollment.

The 31 benefits are divided into 4 blocks: Academia, Sport, Health and Others, as described by Hernando et al. (40). In Table 3 we observe that scholarships are considered the most important benefit, followed by changes in exam dates and justification of absences. The least important benefits include having a separate academic group, specific courses, and taking semesters off.

On the other hand, the most used benefits are the justification of absences, changes in exam dates, and the option to choose a class group. The least used benefits are having semesters off, taking specific courses and having an adapted catering service.

The applied analysis enables us to understand the importance that DC students attach to a benefit offered by the university and, in addition, determine whether they use this benefit. With this dual perspective, the benefits can be ranked by combining both results and classifying the benefits according to both variables: the level of importance and the number of students who use it. Thus, the benefits can be ranked and the universities can identify how best to support DC students by providing them with the most important and most used benefits. The ranking resulting from combining these two variables differs from the individual ranking based on only one of the two criteria. Therefore, in the final ranking, we find that justification for absences, changes in exam dates, and scholarships are the top three benefits, while adapted catering, specific courses and free semesters rank lowest. This criterion for analysis has not been used in previous works (13, 30, 39), so this study provides a new objective approach that facilitates decision-making by higher education institutions.

In this dual-perspective analysis, the question is: if DC students think a benefit is important, do they use it? To answer this, an analysis correlating both variables was conducted. The result showed a positive, strong, and significant correlation, suggesting that Spanish students are very consistent in their choices and that they tend to use what they consider very important, when it is offered to them. The correlation was found to be 0.64 compared to a value of 0.74 in the work by Hernando et al. (40). Although both results are high, their difference may be due to the fact that the list of the 31 benefits originates from a project involving five universities in Europe, which have a different context and idiosyncrasy. As a result, conducting the survey on these benefits exclusively in one country reduces the degree of correlation between the two variables. Nevertheless, it is important to emphasize that students take advantage of what is offered to them and what is important to them. A clear and comparable example is the benefit of scholarships: in Spain, scholarships have the highest importance value and yet are not the most used, perhaps because not all universities offer this benefit to DC athletes. This contrast is even more striking when we analyze the case of European universities in Hernando et al. (40), where scholarships are the third most important benefit, but, when the two criteria are combined, scholarships rank fourteenth because their use is low, indicating that few universities have a scholarship program for DC students. Thus, this analysis provides universities with objective and adjusted criteria for more effective decision-making.

**Figure 6:**
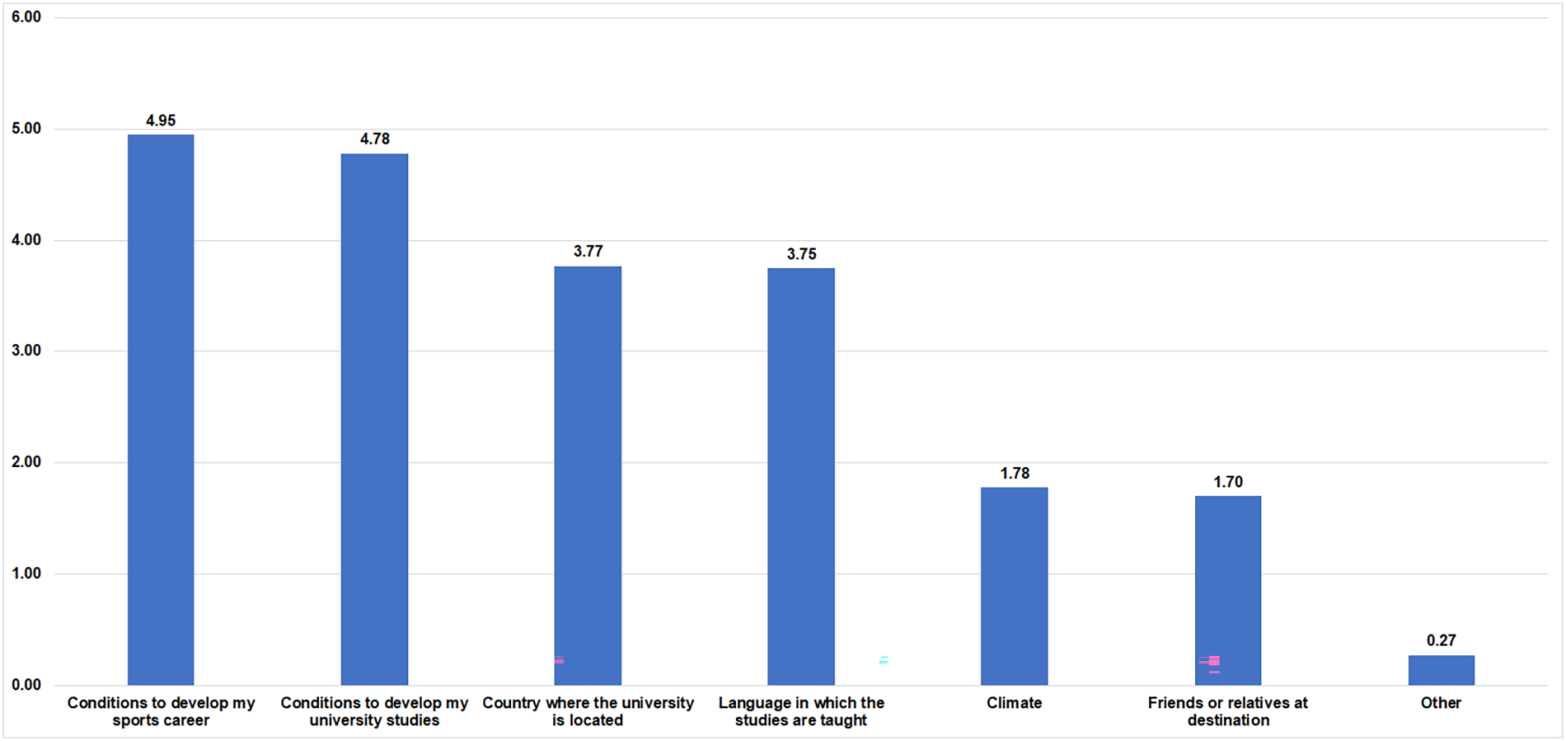
Criteria to choose a university for an Erasmus+ exchange.

Finally, it is important to highlight the interest of DC students in participating in a mobility program within the Erasmus+ program. Eighty-four percent of DC students have expressed their interest in this international mobility. This interest is reflected in the work of Fuchs et al. (57), which involves students from five different countries, and in Ryba et al. (58), which analyzes the profiles, interests and problems associated with transnational mobility. Spanish students also show this interest, knowing that this predisposition is conditioned by two very decisive elements: the continuity of their sporting career and, closely following this, the continuity of their academic career (Figure 6). The other selection criteria are less representative when choosing a university for transnational mobility. These two very important conditions for DC students show their commitment to their dual career. It is one of the great strengths of this group, so any action by the Spanish university to support students in balancing these two facets will help to create a better national and European citizenship and improve their sports and academic career.

The main limitation of this study is the sample collected. The fact that 411 valid responses were obtained from 27 Spanish universities out of the 78 represented in the Spanish University Sports Committee introduces a small bias that limits the generalizability of the conclusions for Spanish universities. Although 563 responses were received, not all of them could be processed because they were not sufficiently completed. In future research, it would be beneficial to obtain more input from the expert personnel on dual career in Spanish universities and to schedule the period of availability of the study, taking into account the specific casuistry of Spanish universities. This approach could potentially enhance the sample size and yield results with greater statistical significance.

The continuation of lines of research that will delve into various aspects in Spanish universities, such as adapted sports, mobility programs, and labor market insertion, will contribute to the advancement of knowledge in these fields. As previously stated, an expansion of the sample would be advantageous for the collection of more comprehensive data and the examination of specific details, such as the number of dual-career athletes who engage in adapted sports modalities and the unique challenges they face in balancing their academic and athletic pursuits. Furthermore, it would be beneficial to ascertain whether dual career students engage in mobility programs during their university studies and, if so, how they reconcile this with their participation in sports. It would also be valuable to understand their experiences, the benefits they perceive, and how they manage to maintain their academic and sports performance. It would also be beneficial to investigate how DC athletes position themselves professionally in the labor market and the advantages they gain from pursuing a dual career trajectory for their professional advancement.

## 5 Conclusions

It can be affirmed that there is a specific identity among student-athletes' dual career in Spanish universities, which necessitates a set of measures to help them balance the demands of combining their sports career with their academic career.

In Spain, the government acts as a regulator and facilitator, enabling DC student athletes to access and combine their studies with their sports careers. However, it is the Spanish universities, within the framework of their autonomy, that are responsible for implementing various actions to develop and expand programs for elite athletes, both in terms of benefits and access criteria to their own programs.

This document enables the identification of the aspects deemed most pertinent to the student, the resources they utilize and the obstacles they encounter. The outcome is a pertinent instrument that can be employed by the university to incorporate actions within its benefit program for DC students that are most impactful to them. For instance, the provision of scholarships for their particular expenses and the accessibility of sports facilities at no cost facilitate the reduction of travel and enhance time management for the student athlete, a significant challenge within this demographic.

There is a significant number of elite athletes pursuing higher education, with a balance between men and women, spanning a wide range of sports disciplines. Notably, some DC student athletes participate in adapted sports. The establishment of a dedicated structure for DC athletes within the university provides a centralized point of reference for them, offering guidance, support, and assistance in navigating the requirements of the elite athlete program as approved by the university's governing bodies.

A new methodology has been developed to assess the benefits offered by universities to DC students. This method combines the students' opinions on the importance of the benefit and its usage, resulting in a ranking with dual criteria that facilitate decision-making when a university decides to include or promote a benefit. There is a positive, strong and significant relationship between the perceived importance of a benefit and its use —a relationship that can be further strengthened if universities tailor these benefits to meet the needs of student athletes.

It should be noted that these DC students demonstrate a high level of interest in transnational mobility and that their main concerns being the continuity of their sports and academic careers, underscoring their commitment to both.

## Data Availability

The datasets presented in this article are not readily available because the datasets presented in this article are not readily available because the traceability of responses can lead to the identification of the subjects. Partial data are available upon request to the corresponding author. Requests to access the datasets should be directed to Carlos Hernando, hernando@uji.es.
