# Supplementary material for: Dual-career student athletes in Spanish universities: characteristics and interests
Source: Front Sports Act Living. 2025 Jan 6;6:1507859. doi: 10.3389/fspor.2024.1507859 (PMC11743630; doi:10.3389/fspor.2024.1507859)
Supplement: Supplementary file 1 [file Table1.docx]

**Supplementary Material 1**

Q1 – Consent

This survey is part of a programme entitled Student Athletes Erasmus+ Mobility in Europe (SAMEurope), which is being carried out by the Institut national des sciences appliqués de Lyon (INSA Lyon), Karlsruhe Institute of Technology (KIT), Chalmers University of Technology (Chalmers), Jyväskylä University (JYU) and Universitat Jaume I (UJI). Its main objective is to implement the recommendations of the European Union on the dual career (DC) of high-level student athletes and promote their inclusion in the Erasmus + mobility program. The confidential data obtained will be used for a research project that will focus on the dual career of university students.

The principal researcher of this project is Carlos Hernando Domingo. If you have any questions about the project, or need any clarification, please send a message in English to: hernando@uji.es. This email is sent to you as a dual career university student. To participate in this study, we ask you to answer the questionnaire below. It is a simple online questionnaire of 27 questions, which will take you about 15 minutes to answer. In the questionnaire you will have to answer questions about your dual career and the way in which you combine your university studies with your sports career.

Your participation is very important, as your answers will help us to understand the needs of the group of high-level student athletes in Europe who follow a dual career. This knowledge will make it possible to better adjust the conditions for these students and open the door to the possibility of generating a specific mobility programme within the Erasmus+ programme. Your participation is voluntary and does not entail any remuneration. If you want to withdraw from the study once it has started, you can do so by sending an email to the principal researcher.

The final results will be published by the consortium of the five universities, once they have been delivered to the European Commission. This research has been approved by the Ethics Committee of the Universitat Jaume I (CEISH/27/2022) and complies with the guidelines of the Declaration of Helsinki. The responses to this survey constitute personal data and, therefore, their processing is subject to the provisions of the relevant regulation on this matter. In particular, the General Data Protection Regulation and Organic Law 3/2018, of December 5, on the Protection of Personal Data and guarantee of digital rights.

We need you to provide us with your consent for the collection and subsequent processing of your data, which we will carry out in accordance with the following description:

Responsible for the treatment

Universitat Jaume I, Sports Service

Purpose of the treatment

Study of the application of the recommendations of the EU Guidelines on the dual career of athletes (2012) on the European dimension of dual careers in sport, with the aim of improving the inclusion of dual career students in the program Erasmus+.

Legitimation

Consent

Organic Law 6/2001, of December 21, on Universities.

Recipients

No data will be transferred to anyone other than the members of the research team.

Rights

You can exercise your rights of access, rectification, deletion and portability, and the limitation or opposition to the treatment before the General Secretariat of the Universitat Jaume I through the Electronic Registry (https://ujiapps.uji.es/reg/rest/publicacion /generic_request) or, in person, at the Information and Registration Office (InfoCampus), located in the Ágora Universitaria - Locales 14-15.

Additional information

You can consult additional and detailed information on this data processing at Information <https://www.uji.es/protecciodades/clausules/?t=I164>

- I agree to participate
- I do not agree to participate

Q2 – Year of birth

| 2005 | 2002 | 1999 | 1996 | 1993 | 1990 | 1987 | 1984 |
| --- | --- | --- | --- | --- | --- | --- | --- |
| 2004 | 2001 | 1998 | 1995 | 1992 | 1989 | 1986 | 1983 |
| 2003 | 2000 | 1997 | 1994 | 1991 | 1988 | 1985 | 1982 |

Q3 – Gender

- Woman
- Men
- Non-binary

Q4 – Country in which you are studing

- Germany
- Spain
- Finland
- France
- Sweden

Q5 – Name of the university where you study

- Universidad Antonio de Nebrija
- Universidad Carlos III de Madrid
- Universidad Católica de Ávila
- Universidad Católica San Antonio de Murcia
- Universidad Católica San Vicente Mártir de Valencia
- Universidad de Alcalá
- Universidad de Alicante
- Universidad de Almería
- Universidad de Barcelona
- Universidad de Burgos
- Universidad de Cádiz
- Universidad de Castilla La Mancha
- Universidad de Deusto
- Universidad de Extremadura
- Universidad de Girona
- Universidad de Granada
- Universidad de Huelva
- Universidad de las Islas Baleares
- Universidad de Las Palmas de Gran Canaria
- Universidad de Málaga
- Universidad de Mondragón
- Universidad de Murcia
- Universidad de Navarra
- Universidad de Salamanca
- Universidad de Sevilla
- Universidad de Valencia
- Universidad de Valladolid
- Universidad de Vigo
- Universidad de Zaragoza
- Universidad del País Vasco
- Universidad Europea del Atlántico
- Universidad Isabel I
- Universidad Miguel Hernández
- Universidad Nacional de Educación a Distancia (UNED)
- Universidad Pablo de Olavide
- Universidad Politécnica de Cartagena
- Universidad Politécnica de Madrid
- Universidad Pública de Navarra
- Universidad Ramon Llull
- Universidad San Pablo CEU
- Universitat Abat Oliba CEU
- Universitat Autònoma de Barcelona
- Universitat Internacional de Catalunya (UIC Barcelona)
- Universitat Jaume I de Castelló
- Universitat Politècnica de València
- Universitat Pompeu Fabra
- Universitat Rovira i Virgili

Q6 – Level of studies

- Bachelor’s
- Master’s
- PhD

Q7 – Branch of knowledge

- Logic
- Mathematics
- Astronomy and astrophysics
- Physics
- Chemistry
- Life Sciences
- Earth and Space Sciences
- Agricultural Sciences
- Medical Sciences
- Technological Sciences
- Anthropology
- Demographics
- Economic Sciences
- Geography
- History
- Juridical Sciences and Law
- Linguistics
- Pedagogy
- Political Science
- Psychology
- Science of Arts and Letters
- Sociology
- Ethics
- Philosophy

Q8 – Name of the degree you are pursuing

Q9 – Number of credits enrolled in this academic year

Q10 – Hours of dedication per week to university studies (class hours and autonomous work)

Q11 – Type of sport you compete in

- Adapted
- Not adapted

Q12 – Sports discipline

| Aeronautics | Alpine skiing | Archery | Artistic gymnastics | Badminton |
| --- | --- | --- | --- | --- |
| Bandy / Ball Hockey | Baseball and softball | Basketball | Beach volleyball | Boxing |
| Canoeing | Cheerleading | Chess | Climbing | Cross-country running |
| Cross-country skiing | Cycling | Dance Sport | Downhill MTB | Equestrian |
| Fencing | Floorball | Football | Frisbee | Frontenis |
| Golf | Handball | Field Hockey | Ice Hockey | Indoor football |
| Judo | Jujitsu | Karate | Kayak | Kickboxing |
| Lifesaving and lifeguarding | Modern Pentathlon | Motorcycling | Motorsport | Olympic shooting |
| Olympic wrestling | Orienteering | Paddle | Petanque | Rhythmic gymnastics |
| Rowing | Rugby | Sailing | Sambo | Skateboard |
| Skating | Squash | Surfing | Swimming | Synchronized swimming |
| Table tennis | Taekwondo | Tennis | Track and field | Trail |
| Triathlon | Underwater activities | Valencian Pelota | Volleyball | Water skiing |
| Weightlifting | Other |  |  |  |

Q13 – Sport level

- Regional
- National
- International

Q14 – Highest international championship you have competed in

- Olympic Games
- World Championship
- European Championship
- International University Championship
- National Team

Q15 – Hours of dedication per week to sport (training, competitions, physiotherapy, coaching, etc.)

Q16 – Do you participate in university competitions?

- Yes
- No

Q17 – Which criteria did you meet to enter university?

- Academic criteria (cut-off mark)
- Sport criteria (places reserved for elite athletes)

Q18 – Have you chosen your university because it offers support to combine your studies and your sporting activity?

- Yes
- No

Q19 – Rate from 0 to 5 the problems you have when combining elite sport with studies (0 = not an important problem, 5 = a very important problem)

|  | 0 | 1 | 2 | 3 | 4 | 5 |
| --- | --- | --- | --- | --- | --- | --- |
| Long absence from classes due to competitions / training camps |  |  |  |  |  |  |
| Occasional absence from classes due to training sessions |  |  |  |  |  |  |
| Absence from university tests and exams due to competitions / training camps |  |  |  |  |  |  |
| Time limit to finish university studies |  |  |  |  |  |  |
| Reduced training sessions due to university studies |  |  |  |  |  |  |
| Financial uncertainty |  |  |  |  |  |  |
| Time spent commuting |  |  |  |  |  |  |
| Little leisure time |  |  |  |  |  |  |

Q20 – Do you know how your university may support you to combine university studies and high-level sports?

- Yes
- No

Q21 – Rate from 0 to 5 the importance of each of these benefits in order to be able to combine your university studies and your high-level sport (0 = not at all important, 5 = very important)

|  | 0 | 1 | 2 | 3 | 4 | 5 |
| --- | --- | --- | --- | --- | --- | --- |
| Choose class groups |  |  |  |  |  |  |
| Justification of absences |  |  |  |  |  |  |
| Online courses |  |  |  |  |  |  |
| Remedial courses |  |  |  |  |  |  |
| Specific courses (eg time management, sports marketing & social media) |  |  |  |  |  |  |
| Changing exam dates |  |  |  |  |  |  |
| Online exams (without changing dates) |  |  |  |  |  |  |
| Adaptation of the pace of study |  |  |  |  |  |  |
| Extension of number of exam session |  |  |  |  |  |  |
| Extension of the criteria for permanency |  |  |  |  |  |  |
| Partial enrollment |  |  |  |  |  |  |
| Free semesters |  |  |  |  |  |  |
| Separate academic group |  |  |  |  |  |  |
| Academic tutoring |  |  |  |  |  |  |
| Career advice |  |  |  |  |  |  |
| Free use of sports facilities |  |  |  |  |  |  |
| Private use of sports facilities |  |  |  |  |  |  |
| Reservation of places for sports courses |  |  |  |  |  |  |
| Extra credit for participation in university sport events |  |  |  |  |  |  |
| DC tutoring |  |  |  |  |  |  |
| General medical services |  |  |  |  |  |  |
| Mental health support |  |  |  |  |  |  |
| Physiotherapy |  |  |  |  |  |  |
| Nutritionist |  |  |  |  |  |  |
| Testing (physiology, biomechanics, performance) |  |  |  |  |  |  |
| Specialized PE teachers |  |  |  |  |  |  |
| Housing |  |  |  |  |  |  |
| Discounts in meals |  |  |  |  |  |  |
| Adapted catering service |  |  |  |  |  |  |
| Scholarships |  |  |  |  |  |  |
| Extra points in Erasmus evaluation |  |  |  |  |  |  |

Q22 – Have you used any of the benefits offered by your university to combine your university studies and your high-level sport?

- Yes
- No

Q23 – Indicate which Benefit or benefits you have used

| Choose class groups | Justification of absences |
| --- | --- |
| Online courses | Remedial courses |
| Specific courses (eg time management, sports marketing & social media) | Changing exam dates |
| Online exams (without changing dates) | Adaptation of the pace of study |
| Extension of number of exam session | Extension of the criteria for permanency |
| Partial enrollment | Free semesters |
| Separate academic group | Academic tutoring |
| Career advice | Free use of sports facilities |
| Private use of sports facilities | Reservation of places for sports courses |
| Extra credit for participation in university sport events | DC tutoring |
| General medical services | Mental health support |
| Physiotherapy | Nutritionist |
| Testing (physiology, biomechanics, performance) | Specialized PE teachers |
| Housing | Discounts in meals |
| Adapted catering service | Scholarships |
| Extra points in Erasmus evaluation |  |

Q24 – Rate from 0 to 5 who supports you in your dual career at the university level (0 = not at all, 5 = a lot)

|  | 0 | 1 | 2 | 3 | 4 | 5 |
| --- | --- | --- | --- | --- | --- | --- |
| Faculty or academic staff |  |  |  |  |  |  |
| Administrative staff or service staff |  |  |  |  |  |  |
| Classmates |  |  |  |  |  |  |
| Other people |  |  |  |  |  |  |

Q25 – Would you like to do an Erasmus exchange?

- Yes
- No

Q26 – Organise the following criteria according to their importance when choosing a university for an Erasmus exchange

| Conditions to develop my sports career |
| --- |
| Conditions to develop my university studies |
| Country where the university is located |
| Language in which the studies are taught |
| Friends or relatives at destination |
| Climate |
| Other |

Q27 – Do you feel that your university recognizes your sports career?

- Yes
- No

QFinal – If you want to receive information about the project and its results, please write your email:

SAMEurope, Student Athletes Erasmus+ Mobility in Europe, is a project Funded by the European Union. Views and opinions expressed are however those of the author(s) only and do not necessarily reflect those of the European Union or EACEA. Neither the European Union nor the granting authority can be held responsible for them.

We thank you for your time spent taking this survey. Your response has been recorded.
