# Supplementary material for: Dual-career student athletes in Spanish universities: characteristics and interests
Source: Front Sports Act Living. 2025 Jan 6;6:1507859. doi: 10.3389/fspor.2024.1507859 (PMC11743630; doi:10.3389/fspor.2024.1507859)
Supplement: Supplementary file 2 [file Table2.docx]

**Supplementary Material 2**

Number and percentage of students by field of knowledge

| FIELD OF KNOWLEDGE | RESPONSES | PERCENTAGE |
| --- | --- | --- |
| Medical Sciences | 102 | 24.82% |
| Technological Sciences | 66 | 16.06% |
| Economic Sciences | 58 | 14.11% |
| Life Sciences | 51 | 12.41% |
| Juridical Sciences and Law | 40 | 9.73% |
| Pedagogy | 26 | 6.33% |
| Psychology | 12 | 2.92% |
| Physics | 11 | 2.68% |
| Mathematics | 9 | 2.19% |
| Chemistry | 8 | 1.95% |
| Linguistics | 8 | 1.95% |
| Science of Arts and Letters | 7 | 1.70% |
| Sociology | 4 | 0.97% |
| Earth and Space Sciences | 3 | 0.73% |
| Logic | 2 | 0.49% |
| Agricultural Sciences | 2 | 0.49% |
| Ethics | 1 | 0.24% |
| Philosophy | 1 | 0.24% |
| Astronomy and astrophysics | 0 | 0.00% |
| Anthropology | 0 | 0.00% |
| Demographics | 0 | 0.00% |
| Geography | 0 | 0.00% |
| History | 0 | 0.00% |
| Political Sciences | 0 | 0.00% |
