# Supplementary material for: Dual-career student athletes in Spanish universities: characteristics and interests
Source: Front Sports Act Living. 2025 Jan 6;6:1507859. doi: 10.3389/fspor.2024.1507859 (PMC11743630; doi:10.3389/fspor.2024.1507859)
Supplement: Supplementary file 3 [file Table3.docx]

**Supplementary Material 3**

Sport discipline

| SPORT DISCIPLINE | RESPONSES | PERCENTAGE |
| --- | --- | --- |
| Track and field | 70 | 17.03% |
| Basketball | 20 | 4.87% |
| Handball | 20 | 4.87% |
| Swimming | 20 | 4.87% |
| Skating | 18 | 4.38% |
| Football | 17 | 4.14% |
| Volleyball | 16 | 3.89% |
| Indoor football | 13 | 3.16% |
| Taekwondo | 13 | 3.16% |
| Rugby | 12 | 2.92% |
| Canoeing | 11 | 2.68% |
| Karate | 11 | 2.68% |
| Equestrian | 8 | 1.95% |
| Field Hockey | 8 | 1.95% |
| Ice Hockey | 8 | 1.95% |
| Sailing | 8 | 1.95% |
| Inline Hockey | 8 | 1.95% |
| Judo | 7 | 1.70% |
| Water polo | 7 | 1.70% |
| Chess | 6 | 1.46% |
| Climbing | 6 | 1.46% |
| Cycling | 6 | 1.46% |
| Triathlon | 6 | 1.46% |
| Fencing | 5 | 1.22% |
| Lifesaving and lifeguarding | 5 | 1.22% |
| Roller hockey | 5 | 1.22% |
| Orienteering | 4 | 0.97% |
| Tennis | 4 | 0.97% |
| Trampoline gymnastics | 4 | 0.97% |
| Olympic shooting | 3 | 0.73% |
| Paddle | 3 | 0.73% |
| Rowing | 3 | 0.73% |
| Squash | 3 | 0.73% |
| Table tennis | 3 | 0.73% |
| Trail | 3 | 0.73% |
| Alpine skiing | 2 | 0.49% |
| Badminton | 2 | 0.49% |
| Downhill MTB | 2 | 0.49% |
| Frontenis | 2 | 0.49% |
| Olympic wrestling | 2 | 0.49% |
| Rhythmic gymnastics | 2 | 0.49% |
| Surfing | 2 | 0.49% |
| Weightlifting | 2 | 0.49% |
| Wushu | 2 | 0.49% |
| Curling | 2 | 0.49% |
| Basque Pelota | 2 | 0.49% |
| Archery | 1 | 0.24% |
| Artistic gymnastics | 1 | 0.24% |
| Bandy / Ball Hockey | 1 | 0.24% |
| Beach volleyball | 1 | 0.24% |
| Cross-country running | 1 | 0.24% |
| Cross-country skiing | 1 | 0.24% |
| Golf | 1 | 0.24% |
| Kayak | 1 | 0.24% |
| Motorsport | 1 | 0.24% |
| Sambo | 1 | 0.24% |
| Synchronized swimming | 1 | 0.24% |
| Underwater activities | 1 | 0.24% |
| Valencian Pelota | 1 | 0.24% |
| Kayak polo | 1 | 0.24% |
| Hunting/Agility | 1 | 0.24% |
| Fishing | 1 | 0.24% |
| Beach tennis | 1 | 0.24% |
| Paddle Surf | 1 | 0.24% |
| Wakeboard | 1 | 0.24% |
| Cricket | 1 | 0.24% |
| Speed skating | 1 | 0.24% |
| Aerobic gymnastics | 1 | 0.24% |
| American football | 1 | 0.24% |
| Windsurfing | 1 | 0.24% |
| Other (not specified) | 1 | 0.24% |
